# Acupuncture as an adjunctive therapy for sleep fragmentation in Parkinson’s disease: a pilot study based on polysomnography

**DOI:** 10.3389/fneur.2025.1550250

**Published:** 2025-03-21

**Authors:** Jili Sheng, Yingying Sun, Tao Liu, Jianfang Zhu, Qinhong Zhang, Xiaoqing Jin

**Affiliations:** ^1^The Second Clinical Medical College of Zhejiang Chinese Medical University, Hangzhou, China; ^2^Department of Acupuncture, Zhejiang Hospital, Hangzhou, China; ^3^Department of Rehabilitation, Ningbo Zhenhai Longsai Hospital, Ningbo, China; ^4^Shenzhen Frontier in Chinese Medicine Research Co., Ltd., Shenzhen, China

**Keywords:** acupuncture, Parkinson’s disease, polysomnography, sleep fragmentation, sleep disorder

## Abstract

**Objective:**

This pilot study aims to assess the feasibility and preliminary effectiveness. of acupuncture as an adjunct therapy for improving sleep quality in patients with Parkinson’s Disease (PD) who are experiencing fragmented sleep disorders.

**Method:**

This pilot study recruited a small cohort of 11 patients diagnosed. With PD, each undergoing a structured 4-week acupuncture intervention comprising three sessions per week. Outcome measures included polysomnography (PSG) and the Pittsburgh Sleep Quality Index (PSQI), both of which were evaluated at baseline and following the completion of the 4-week acupuncture regimen.

**Result:**

Post-intervention analysis showed trends toward improved sleep continuity with statistical significance in the sleep arousal index (*p* = 0.001), sleep arousal frequency (*p* = 0.001), and PSQI scores (*p* = 0.026) compared to baseline measurements. Importantly, no adverse events or complications were reported throughout the study period.

**Conclusion:**

The results indicate that acupuncture provides preliminary evidence supporting its use as a feasible adjunctive therapy for improving sleep quality in individuals with PD. Further research is required to evaluate the long-term efficacy of acupuncture and to examine its practicality and feasibility for integration into established PD management protocols.

## Introduction

Fragmented sleep is a common and debilitating condition affecting individuals with Parkinson’s disease (PD), significantly diminishing their quality of life. It is characterized by frequent awakenings and disrupted sleep architecture, which exacerbate both motor and non-motor symptoms of PD, including cognitive decline, mood disturbances, and reduced overall functionality (1, 2). The diagnosis of fragmented sleep typically relies on standardized assessments such as polysomnography (PSG), which evaluates key metrics, including the sleep arousal index, defined as the number of microarousals per hour (3). This index serves as a primary indicator of sleep fragmentation severity (4).

Existing treatment strategies for sleep fragmentation, including pharmacological interventions and behavioral therapies, often have limitations such as side effects and suboptimal efficacy in addressing sleep disturbances (5). Emerging evidence suggests that acupuncture may serve as an effective adjunctive therapy for individuals with PD experiencing fragmented sleep. Studies have demonstrated that acupuncture can improve sleep quality and reduce sleep disturbances in diverse populations, supporting its potential as a complementary therapeutic approach (6–8). However, the specific effects of acupuncture on sleep disturbances within the PD population remain underexplored, highlighting the need for further research in this area.

This pilot study aims to evaluate the feasibility and preliminary effectiveness of acupuncture as an adjunctive therapy for fragmented sleep in individuals with PD. The findings are anticipated to inform future studies and support the potential integration of acupuncture into standardized therapeutic protocols for managing sleep disturbances in PD.

## Materials and methods

### Study design

This pilot study was conducted at the Outpatient Department of Acupuncture at Zhejiang Hospital (Medical Ethics Approval No. 2019-29K) from May 2024 to November 2024, with the aim of evaluating the effects of acupuncture on sleep disturbances in patients with PD. A total of 11 patients diagnosed with PD, all of whom reported experiencing sleep disturbances, were enrolled in the study. Each participant underwent a standardized 4-week acupuncture protocol, which included of three sessions per week.

### Eligibility criteria

#### Inclusion criteria

Participants must meet the following inclusion criteria for the study: (1) A confirmed diagnosis of PD, regardless of age or gender, with no alternative therapy received in the month prior to enrollment. (2) The presence of fragmented sleep disorder, indicated by an arousal index exceeding 10. The arousal index is defined as the frequency of brief awakenings or disruptions in sleep, with a higher index indicating poorer sleep quality and continuity. (3) Documented stable use of sleep-related medications (benzodiazepines, non-benzodiazepine hypnotics, or melatonin) with unchanged regimens maintained for at least 4 weeks prior to enrollment and throughout the study. (4) Comprehensive demographic and clinical data, including age, gender, disease duration, and other pertinent medical information, must be available for review. (5) Participants should be capable of adhering to acupuncture treatment protocols, scale assessments, and PSG monitoring. Additionally, they must provide informed consent, including consent for the public dissemination of their scale and PSG data.

#### Exclusion criteria

Participants will be excluded from the study if they meet any of the following criteria: (1) The presence of other severe neurological disorders that may interfere with the study’s outcomes or complicate the interpretation of the results. (2) Known contraindications to acupuncture treatment, such as allergies to needles or specific acupuncture points, or any medical conditions that contraindicate acupuncture. (3) Inability to complete the full cycle of acupuncture treatment, as determined by non-compliance or other medical issues preventing participation. (4) Severe cognitive impairment, indicated by a Mini-Mental State Examination (MMSE) score of less than 10, or language disorders that significantly hinder the participant’s ability to complete the assessment scales. (5) Recent (≤4 weeks) initiation or changes in sleep medications or use of investigational sleep aids.

### Intervention

Acupuncture was introduced as an adjunctive therapy to the patients’ existing treatment regimens, with no changes made to their sleep medication or dosage during the four-week intervention period. The selected acupoints for the intervention included Baihui (GV20), Yintang (DU29), Shenmen (HT7), Sanyinjiao (SP6), Taichong (LR3), Zusanli (ST36), Hegu (LI4), Zhongwan (CV12), Xiawan (CV10), Qihai (CV6), Guanyuan (CV4), and Huaroumen (ST24). The intervention utilized sterile, disposable filament needles (φ0.25 × 40 mm; Lejiu, China), and all procedures were conducted by a physician trained in acupuncture. The treatment lasted for 4 weeks, with each session lasting 30 min during which the needles were retained. Sessions were conducted three times per week.

### Assessment procedure

Throughout the study, all patients underwent regular clinical evaluations and received routine care. Sleep data were collected at baseline and after the intervention using polysomnography (PSG; Philips Alice 5, USA). Specific PSG measures included sleep efficiency, percentage of wakefulness, percentage of rapid eye movement (REM) sleep, and non-rapid eye movement stages 1 (N1), 2 (N2), and 3 (N3) sleep. Additionally, the apnea-hypopnea index, number of leg movements, and leg movement index were assessed. Clinical assessments included the motor function section of the Unified Parkinson’s Disease Rating Scale (UPDRSIII), the Pittsburgh Sleep Quality Index (PSQI), the modified Parkinson’s Disease Sleep Scale-2 (PDSS-2), and the Epworth Sleepiness Scale (ESS). Each of these scales serves as a standardized tool for assessing various aspects of Parkinson’s disease and its impact on daily life. The UPDRSIII specifically focuses on motor function, with scores ranging from 0 to 56; higher scores indicate greater severity of motor impairments (9). The PSQI is a self-rated questionnaire designed to measure sleep quality and disturbances over a one-month period. The total PSQI score ranges from 0 to 21, with a score of 5 or greater indicating poor sleep quality. Higher scores reflect more severe sleep disturbances and greater daytime impairment (10). The modified PDSS-2 is a 15-item questionnaire specifically designed to evaluate sleep problems in patients with Parkinson’s disease. The total PDSS-2 score ranges from 0 to 60, with higher scores indicating more severe sleep disturbances. A score of ≥18 serves as a screening threshold, defining clinically relevant PD-specific sleep disturbances (11, 12). The ESS is a simple, eight-item questionnaire used to evaluate daytime sleepiness. The total ESS score ranges from 0 to 24, with a score of 10 or more considered indicative of excessive daytime sleepiness. This scale is particularly useful for identifying patients who may benefit from further evaluation or treatment for sleep disorders (13).

### Sample size calculation

The sample size estimation for this pilot study was conducted to provide preliminary data that will inform the design and sample size calculation of a larger definitive trial. In this pilot study, we propose to enroll a total of 20 participants to assess the feasibility, safety, and preliminary efficacy of acupuncture for sleep fragmentation in PD. This sample size will enable us to estimate the variability in the sleep arousal index, sleep arousal frequency, and other key parameters. Additionally, it will allow us to evaluate adherence to the intervention and study protocol, preliminarily assess the safety profile of the intervention, and obtain initial estimates of effect sizes to inform the sample size calculation for a future definitive trial.

### Statistical analysis

Statistical analyses were performed using SPSS software, version 27.0 (IBM Corp., USA). The Shapiro–Wilk test was employed to evaluate the normality of the data distribution. For data following a normal distribution, paired t-tests were conducted, and the results were expressed as mean ± standard deviation. For non-normally distributed data, the Wilcoxon signed-rank test was applied, with results presented as median (interquartile range). *p* < 0.05 was considered statistically significant.

## Results

**Figure 1:**
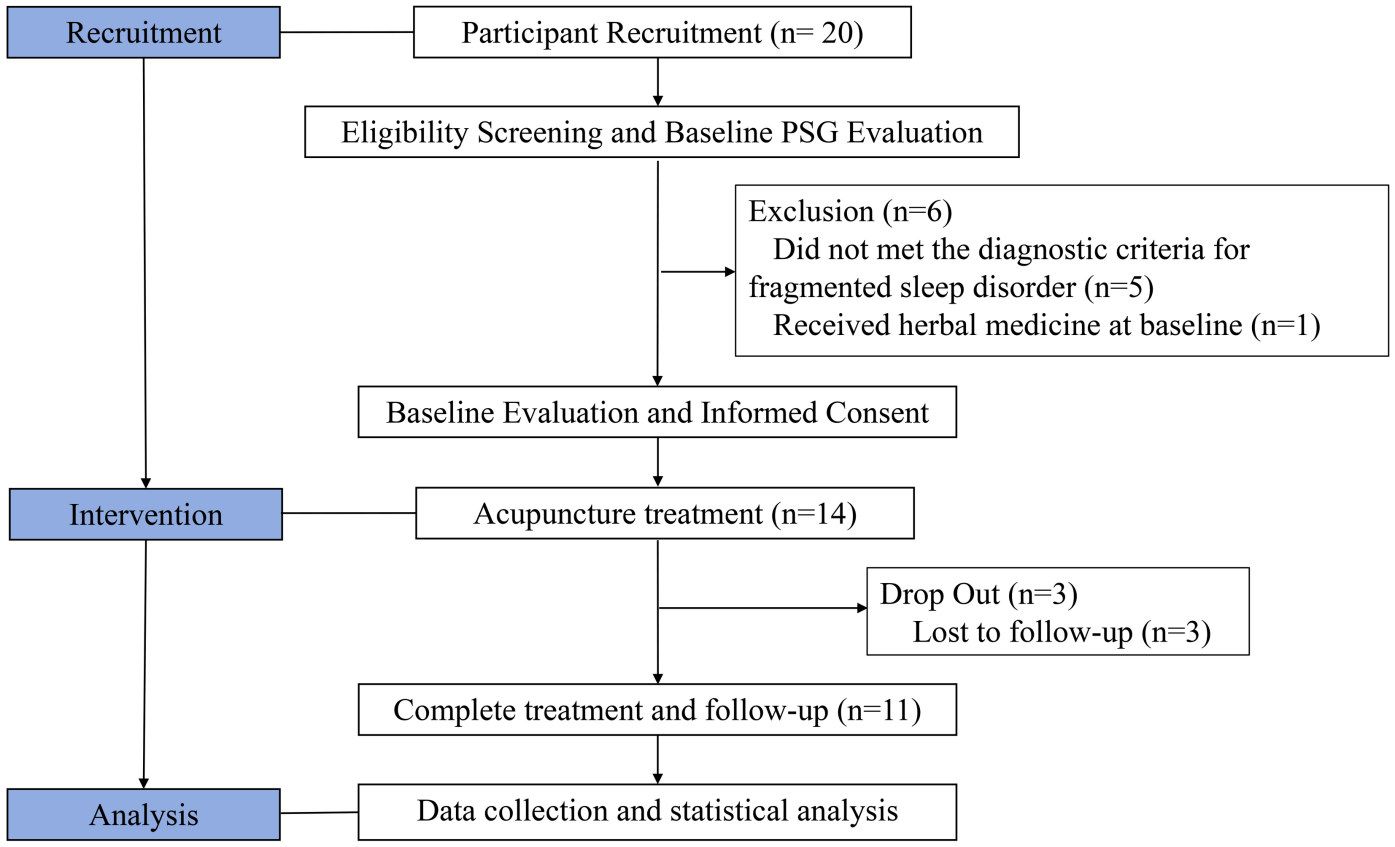
Flowchart of patient selection.

The study selection process is illustrated in Figure 1. Initially, 20 individuals diagnosed with PD were screened and assessed. Of these 6 were excluded: 5 individuals did not met the diagnostic criteria for fragmented sleep disorder and 1 patient was receiving traditional medicine at baseline. Ultimately, 2 participants withdrew from the study, resulting in a final cohort of 11 individuals with complete pre- and post-treatment data available for analysis.

Among the 11 participants, 7 were male and 4 were female, resulting in a male-to-female ratio of 7:4. The mean age of the participants was 67.36 years, with an average MMSE score of 26.37. The median duration of PD and sleep disturbances were 8.41 years and 5.32 years, respectively. The median Levodopa equivalent dose was 500.42 mg/day. PD severity, as assessed using the modified Hoehn and Yahr scale, revealed that one participant was classified at a stage below 2.5, four participants were classified at stage 2.5, and six participants were classified at stage 3 (Table 1).

**Table 1:** Demographic and clinical characteristic information of patients.

Characteristic	PD patients (*n* = 11)
Sex	
Male	7
Female	4
Age, mean (SD), y	67.36 ± 8.38
MMSE	26.37 ± 2.37
Duration of PD, mean (SD), y	8.41 (10.32)
Duration of sleep disoreder, mean (SD), y	5.32 (8.35)
Levodopa equivalent dose, mean (SD), mg/day	500.42 (464.23)
PD severtiy by modified Hoehn and Yahr stage	
<2.5	1
2.5	4
3	6

MMSE, the Mini-Mental State Examination.

Turning to the treatment outcomes illustrated in Table 2, it is important to note that the changes observed in UPDRS-III and ESS did not reach statistical significance, suggesting that the interventions may not have had a measurable impact on these specific aspects of the patients’ conditions. However, the PSQI score demonstrated a significant reduction, decreasing from 5.82 ± 1.60 at baseline to 5.18 ± 1.33 following the intervention (*p* = 0.026), indicating a noteworthy improvement in the patients’ sleep quality after the administered treatment. Furthermore, there was a slight decrease in the scores of PDSS-2, which, while not statistically significant, suggested a potential trend toward enhanced sleep status among the participants, indicating that the treatment may have had some positive effects on their overall sleep experience.

**Table 2:** Effects of scale scores for the treatment of sleep fragmentation in PD.

Outcome measurement	Pre-treatment	Post-treatment	t/Z	*p* value
UPDRSIII	55.34 (25.01)	44.12 (23.24)	−1.585	0.113
PSQI	5.82 ± 1.60	5.18 ± 1.33	2.609	0.026
PDSS-2	30.09 ± 7.80	27.09 ± 8.15	2.084	0.064
ESS	12.36 ± 4.92	11.00 ± 4.20	1.487	0.168

UPDRSIII, the Unified Parkinson’s Disease Rating Scale; PSQI, the Pittsburgh Sleep Quality Index; PDSS-2, the modified Parkinson’s Disease Sleep Scale-2; ESS, the Epworth Sleepiness Scale.

Changes in various sleep parameters including sleep efficiency, percentage of wakefulness, REM sleep, and stages N1, N2 and N3 sleep, as well as the apnea-hypopnea index, number of leg movements, and the leg movement index were not statistically significant before and after treatment. However, significant improvements were observed in the number of microarousals and the arousal index, both of which decreased notably (Table 3). Specifically, the number of microarousals significantly decreased from 217.55 ± 135.51 to 41.45 ± 64.40 (*p* = 0.001), while the arousal index was reduced from 47.40 ± 24.39 to 17.25 ± 23.71 (*p* = 0.001). These findings indicate an improvement in the sleep quality of the patients following treatment, particularly in terms of reduced arousal. Importantly, no adverse events or complications were reported throughout the study.

**Table 3:** Effects of polysomnography for the treatment of sleep fragmentation in PD.

Indicators	Pre-treatment	Post-treatment	t/Z	*p* value
Sleep efficiency (%)	61.97 ± 15.94	60.15 ± 18.99	0.385	0.709
W (%)	38.03 ± 15.93	38.17 ± 20.74	−0.029	0.977
REM (%)	14.53 ± 9.29	13.33 ± 8.75	0.379	0.713
N1 (%)	13.51 (20.62)	22.43 (13.14)	−0.978	0.328
N2 (%)	54.45 ± 22.21	57.54 ± 19.35	−0.891	0.394
N3 (%)	7.49 ± 9.64	7.12 ± 8.63	−3.37	0.876
Number of microarousals	217.55 ± 135.51	41.45 ± 64.40	4.587	0.001
Arousal index (times/h)	47.40 ± 24.39	17.25 ± 23.71	4.567	0.001
AHI (Apnea-hypopnea Index, events/h)	14.14 ± 22.52	15.33 ± 23.17	−0.939	0.370
Number of leg movements	59.00 ± 106.31	47.11 ± 70.96	0.301	0.771
Leg movement Index (times/h)	13.21 ± 21.70	10.39 ± 13.96	0.380	0.714

W, wakefulness; REM, rapid eye movement; N1, non-rapid eye movement stage 1; N2, non-rapid eye movement stage 2; N3, non-rapid eye movement stage 3.

## Discussion

This study underscores the importance of addressing fragmented sleep disorders in individuals with PD, a prevalent condition that profoundly affects quality of life. By highlighting the limitations of existing therapeutic options, this research emphasizes the urgent need for effective and targeted interventions to manage the multifaceted sleep disturbances associated with PD.

Existing literature highlights fragmented sleep disorder as a prevalent feature in PD, contributing to adverse outcomes such as excessive daytime sleepiness, cognitive decline, and diminished overall well-being. Evidence suggests that sleep disturbances often precede the clinical onset of PD and are closely linked to neurodegenerative processes, particularly dopaminergic pathway dysfunction (14, 15). Acupuncture has gained attention as a complementary therapy, with prior studies indicating its efficacy in improving sleep quality across diverse populations (16). In this study, we observed significant reductions in PSQI scores, alongside decreases in the arousal index and the number of microarousals. These findings suggest that acupuncture may have a beneficial effect on subjective sleep quality and sleep continuity. Furthermore, these results are consistent with existing evidence, highlighting the potential role of acupuncture in alleviating sleep disturbances associated with PD.

Although no significant changes were observed in the total scores of the PDSS-2 and ESS, specific items, such as ‘difficulty in maintaining sleep’ from the PDSS-2, demonstrated a trend of improvement following acupuncture treatment. This improvement was consistent with the significant decrease in the number of microarousals and the arousal index. The lack of difference in PDSS-2 and ESS scores before and after treatment may be attributed to the limited number of patients enrolled and the varying nighttime symptoms experienced by different individuals. Existing studies suggest that acupuncture enhances subjective sleep satisfaction by regulating neurotransmitter levels and promoting parasympathetic activation, which may alleviate sleep-related discomfort and consequently improve specific symptoms (17, 18).

The selection of acupoints in acupuncture treatment is grounded in their established regulatory effects on physiological functions, as supported by contemporary medical research. Specifically, the acupoints Baihui (GV20) and Yamen (DU29), located on the head, have been shown to modulate neurotransmitter release and cerebral blood flow. This modulation enhances brain microcirculation and exerts sedative and calming effects (19, 20). The acupoint Shenmen (HT7) has been found to regulate the autonomic nervous system, particularly the balance between the sympathetic and parasympathetic branches. Such regulation can provide cardioprotective and calming effects, thereby alleviating symptoms of palpitations. Additionally, the acupoint Sanyinjiao (SP6) has been shown to modulate the endocrine system and promote blood circulation (21). The acupoint Taichong (LR3) can regulate mood, relieve stress, and mitigate insomnia caused by emotional disorders (22). Furthermore, the acupoints Qihai (CV4) and Guanyuan (CV6) have demonstrated the ability to modulate the endocrine system and enhance immune function (23, 24). Moreover, the acupoints Zusanli (ST36) and Hegu (LI4), along with Zhongwan (CV12) and Xiawan (CV13) from the Conception Vessel, and Tianshu (ST24) from the Stomach Meridian, have been shown to modulate gastrointestinal motility, promote digestion, and alleviate bloating, contributing to improved sleep quality (25–27). In summary, by modulating neurotransmitters, blood circulation, the endocrine system, and gastrointestinal function, these acupoints can effectively improve insomnia and related symptoms.

The safety profile of acupuncture as a treatment modality is particularly noteworthy. In this study, no adverse events or complications were reported, underscoring its safety—a significant advantage compared to the potential side effects associated with pharmacological therapies for PD. Furthermore, the integration of acupuncture into routine care protocols for PD may enhance outcomes by addressing both motor and non-motor symptoms, which collectively have a profound impact on quality of life. This holistic approach aligns with the emerging paradigm of patient-centered care in chronic disease management, emphasizing the necessity for interventions that promote patient engagement and autonomy.

However, this study has several limitations. The small sample size and the absence of a control group restrict the generalizability of the findings. Although the preliminary results suggest that acupuncture may help improve sleep, the efficacy and safety of this intervention cannot be definitively established. Furthermore, the lack of a control group makes it impossible to rule out the influence of placebo effects or other external factors precludes the ability. Therefore, future research should prioritize larger, randomized controlled trials to validate the efficacy of acupuncture in managing sleep disorders in PD. Additionally, exploring the long-term effects of acupuncture and its interactions with other therapeutic modalities could provide valuable insights into its role within a comprehensive management framework for PD.

## Conclusion

The results of this study suggest that acupuncture as potential as an adjunctive therapy for alleviating sleep fragmentation in individuals with PD. Future research should concentrate on assessing the long-term efficacy of acupuncture and investigating its feasibility for integration into standardized management protocols for PD.

## Data Availability

The original contributions presented in the study are included in the article/Supplementary material, further inquiries can be directed to the corresponding authors.
